# CD58 acts as a tumor promotor in hepatocellular carcinoma via activating the AKT/GSK-3β/β-catenin pathway

**DOI:** 10.1186/s12967-023-04364-4

**Published:** 2023-08-12

**Authors:** Chuanzheng Wang, Fei Cao, Jiahao Cao, Zhen Jiao, Yuting You, Yu Xiong, Wenxiu Zhao, Xiaomin Wang

**Affiliations:** Xiamen Key Laboratory of Translational Medical of Digestive System Tumor, Fujian Provincial Key Laboratory of Chronic Liver Disease and Hepatocellular Carcinoma, Zhongshan Hospital of Xiamen University, School of Medicine,, Xiamen University, Xiamen, 361004 People’s Republic of China

**Keywords:** CD58, sCD58, Hepatocellular carcinoma, β-Catenin, GSK-3β, AKT

## Abstract

**Background:**

Hepatocellular carcinoma (HCC) is one of the most prevalent malignancies worldwide because of rapid progression and high incidence of metastasis or recurrence. Accumulating evidence shows that CD58-expressing tumor cell is implicated in development of various cancers. The present study aimed to reveal the functional significance of CD58 in HCC progression and the underlying mechanisms.

**Methods:**

Immunohistochemical staining (IHC), and western blotting were used to detect the expression of CD58 in HCC tissues and cells. The levels of sCD58 (a soluble form of CD58) in the cell supernatants and serum were assessed by ELISA. CCK-8, colony formation, and xenograft assays were used to detect the function of CD58 on proliferation in vitro and in vivo. Transwell assay and sphere formation assay were performed to evaluate the effect of CD58 and sCD58 on metastasis and self-renewal ability of HCC cells. Western blotting, immunofluorescence (IF), TOP/FOP Flash reporter assay, and subcellular fractionation assay were conducted to investigate the molecular regulation between CD58/sCD58 and AKT/GSK-3β/β-catenin axis in HCC cells.

**Results:**

CD58 was significantly upregulated in HCC tissues. Elevation of CD58 expression correlated with more satellite foci and vascular invasion, and poorer tumor-free and overall survival in HCC patients. Higher sCD58 levels were in HCC patients' serum compared to healthy individuals. Functionally, CD58 promotes the proliferation of HCC cells in vitro and in vivo. Meanwhile, CD58 and sCD58 induce metastasis, self-renewal and pluripotency in HCC cells in vitro. Mechanistically, CD58 activates the AKT/GSK-3β/β-catenin signaling pathway by increasing phosphorylation of AKT or GSK3β signaling, promoting expression of Wnt/β-catenin target proteins and TCF/LEF-mediated transcriptional activity. Furthermore, AKT activator SC-79 or inhibitor LY294002 abolished the inhibitory effect of CD58 silencing on the proliferation, metastasis, and stemness of HCC cells.

**Conclusions:**

Taken together, CD58 promotes HCC progression and metastasis via activating the AKT/GSK-3β/β-catenin pathway, suggesting that CD58 is a novel prognostic biomarker and therapeutic target for HCC.

**Graphical Abstract:**

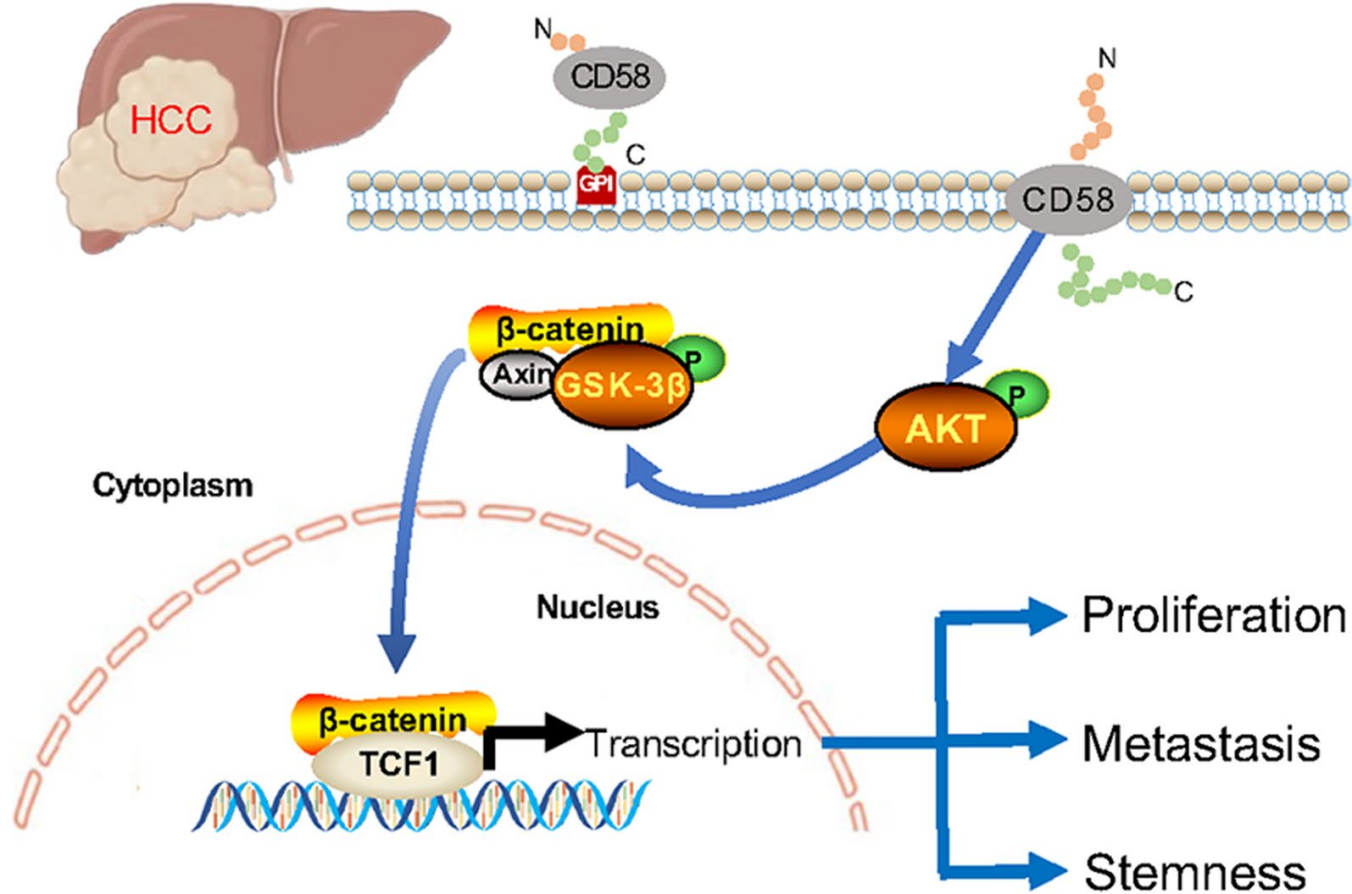

## Introduction

Hepatocellular carcinoma (HCC) is a malignant tumor that is quite prevalent worldwide and is the third leading cause of cancer death [1, 2]. Even after curative resection, the prognosis of HCC patients remains poor due to the high rate of metastatic spread and recurrence [3]. Therefore, there is an urgent need to identify key molecules behind HCC tumorigenesis and progression and elucidate the underlying mechanisms to improve the prognosis of HCC.

CD58 is a highly glycosylated cell surface protein that is expressed in most hematopoietic and non-hematopoietic cells primarily in type I transmembrane or glycosylphosphatidylinositol (GPI)-anchored form [4–6]. CD58 also exists as a soluble form (sCD58) in human serum, urine, and cell supernatant in vitro [7]. As an immune adhesion molecule, CD58 binds to the CD2 receptor on the surface of most natural killer cells and T lymphocytes and engages in immune responses that are critical for cell adhesion and activation [8, 9]. There is growing evidence for the vital role of CD58 in tumors [10, 11]. CD58 is significantly reduced or even absent and associated with poorer overall survival (OS) and disease-free survival in many hematologic malignancies [12, 13]. CD58 loss or downregulation resulted in immune evasion by reducing CTLs and NK cell-mediated cytolysis [14]. However, CD58 has recently been shown to be greatly increased in some solid tumors, such as gastric and colorectal malignancies, and malignant gliomas [15–17]. A recent study reported that CD58 was overexpressed in HCC tissues and positively correlated with tumor-infiltrating immune cells [18]. However, the function and potential mechanisms of CD58 on HCC remained largely elusive.

The Wnt/β-catenin and PI3K/Akt pathways are among the most frequently mutated in cancer [19]. The protein β-catenin, an important element of the Wnt signaling pathway, and phosphorylation of β-catenin by Glycogen synthase kinase (GSK)-3β trigger its degradation in the cytoplasm and reverse the pro-cancer Wnt/β-catenin pathway [20]. While, when GSK-3β is phosphorylated and inhibited by Akt, leading to the accumulation of β-catenin in the cytoplasm, and then β-catenin translocated to the nucleus and interacts with the transcription factor T-cell factor/lymphocyte enhancer factor (TCF/LEF), thereby activating transcription of downstream target genes [21]. Notably, up to 70% of HCC exhibit increased Wnt/β-catenin signaling, and mutations in β-catenin are elevated with HCC progression. Moreover, cooperation between these pathways leads to HCC initiation, progression, invasion, and metastasis [22, 23].

Here, we report that elevated CD58 expression in HCC tissues is correlated with an unfavorable prognosis. CD58 promotes the proliferation of HCC cells in vitro and in vivo. Moreover, CD58 and sCD58 induce metastasis as well as stemness in HCC cells. More importantly, we provide evidence that CD58 and sCD58 exert their pro-tumor effects by activating the AKT/GSK-3β/β-Catenin pathway.

## Materials and methods

### Expression and survival analysis of data from public databases

The correlation between CD58 and survival was determined using data from four databases: TIMER2.0, OncoLnc, GEPIA, and Kaplan–Meier plotter.

### HCC tissue samples, cell lines, animals

All human HCC tissue samples were from the liver cancer biospecimen repository at Zhongshan Hospital, Xiamen University. Before the surgical excision, none of the patients had any additional cancer diagnoses or received anticancer therapy. Telephone interviews and a review of patient’s medical records were used to gather follow-up data. All participants submitted full informed permission, and the Zhongshan Hospital Research Ethics Committee authorized the current research.

The human 293T cell (RRID: CVCL_UE53 and HCC cell lines Huh7 (RRID: CVCL_U443), SK-Hep-1 (RRID: CVCL_0525), MHCC97-L (RRID: CVCL_4973), MHCC-97H (RRID: CVCL_4972), Hep3B (RRID: CVCL_0326), SNU-449 (RRID: CVCL_0454), and PLC/PRF/5 (RRID: CVCL_0485) were acquired from Guangzhou Cellcook Biotechnology Co., Ltd. (Guangzhou, China). By comparing these cell lines to the STR database, these cell lines were verified. In addition to 10% fetal bovine serum (FBS; HyClone, Logan, Utah, USA) and 100 U/ml penicillin–streptomycin (Gibco, Grand Island, USA), SNU-449 cells were grown in RPMI 1640 medium (HyClone, Logan, Utah, USA). In addition to 10% FBS and 100 U/ml penicillin–streptomycin, Dulbecco’s modified Eagle medium (DMEM) was used to support other cell types. Male, 4–6-week-old nude mice were acquired from Charles River Laboratories in Beijing, China, and kept in the Xiamen University Experimental Animal Center under particular pathogen-free conditions. The Xiamen University Animal Protection and Use Committee gave the go-ahead for all experimental methods.

### Reagents and antibodies

Soluble CD58 (sCD58), AKT inhibitor (LY294002), and Akt activator (SC-79) were acquired from Med Chem Express (MCE, Shanghai, China). Anti-Oct4 (11263-1-AP), anti-Sox2 (66411-1-Ig), anti-CD24 (18330-1-AP), anti-vimentin (10366-1-AP), and anti-c-Myc (10828-1-AP) were purchased from Proteintech (Wu Han, China). Anti-EPCAM (#2626S), anti-E-cadherin (#3195S), anti-AKT (#9272S), anti-GSK-3β (#5676S), anti-Phospho-GSK-3β (Ser9) (5558S), anti-β-Catenin (#8480S), anti-non-phosphorylated (active) β-catenin (S33/37/T41) (#8814S), anti-phospho-β-Catenin (Ser552) (#9566S), and anti-cyclin D1 (#55506S) were acquired from Cell Signaling Technology. Anti-CD58 (A0806) and anti-phospho-AKT (Ser473) (AP0140) were purchases from ABclonal (Wu Han, China).

### IHC and staining assessment

IHC was conducted on HCC tissue slices using an anti-CD58 antibody (MA5-29121) from Invitrogen (Shanghai, China). Two qualified pathologists who maintained the confidentiality of the clinicopathological and follow-up data independently conducted staining evaluations. Specimens were scored based on the intensity and degree of staining. The intensity of staining was rated as 0, 1, 2, and 3 (negative, week, strong, moderate, and negative). According to the mean proportion of positively stained cells, the degree of staining was divided into four categories: 0 (0%), 1 (1–25%), 2 (26–50%), and 3 (51–75%) and 4 (76–100%). The final CD58 staining score (0–7) was determined by summing the intensity score and the degree score. This generally straightforward and repeatable scoring procedure has been used in previous research and may provide equal findings when used by different assessors [24]. We classified CD58-High expression as final staining fraction > 5, while CD58-Low expression as other.

### Plasmid construction and transfection

The Public Protein/Plasmid Library (PPL, Jiangsu, China) used target sequences #2 (5′GAAATGGAATCGCCAAATA3′) and #3 (5′GCAGTAAT TACAACATGTA3′) to create CD58 shRNA plasmids and negative control plasmids. The corresponding vector was pPLK-GFP-Puro. Lentiviral expression vectors and control plasmids with psPAX2 and pMD.2G were entered into 293T cells. After 24 h, lentiviral particles were harvested and used to infect HCC cells with 5 μg/ml polybrene (Solarbio, Beijing, China) for 48 h. To generate stably transfected cells, puromycin (5 μg/ml) (Solarbio, Beijing, China) was administered to cells.

### siRNA transfection

We used the following target siRNA sequences for siRNA-mediated CD58: #2 (5′GAAATGGAATCGCCAAATA3′) and #3 (5′GCAGTAATTACAACATGTA3′).

The RNA duplexes were synthesized by RIBOBIO biology (Shanghai, China). Following the manufacturer’s guidelines, siRNA was transfected into HCC cells and human 293T cells with Lipofectamine RNAiMAX Transfection Reagent (Invitrogen, Carlsbad, CA, USA).

### Western blotting

The concentration of protein was measured by a bicinchoninic acid protein assay kit (Invitrogen, Shanghai, China) after cells and tissue samples were processed in RIPA cell lysis buffer (Beyotime, Shanghai, China). The proteins were loaded onto a 10% SDS-PAGE for electrophoresis and then transferred to 0.22 m PVDF membranes (Millipore, MA, USA). Membranes were blocked with blocking buffer (1 × TBST with 5% skim milk), incubated with the appropriate primary antibodies, and treated with the secondary antibody. Immobilon Western Chemiluminescence HRP Substrate (Solarbio, Beijing, China) was then used to visualiza the brands.

### Immunofluorescence staining assay

Coverslips containing cultured cells were fixed in 4% paraformaldehyde, permeabilized with 0.5% Triton X-100 in PBS, blocked with blocking buffer (PBS with 5% bovine serum albumin), and incubated with an anti-non-phosphorylated (active) β-catenin (S33/37/T41) antibody. After treatment with Alexa Fluor 594 secondary antibody, the coverslips were counterstained with DAPI. Laser scanning confocal microscopy (Zeiss LSM780, Carl Zeiss) was used to observe the cells.

### Luciferase reporter assay

293T and HCC cells were co-transfected with TCF/LEF reporter plasmids (Promega, Madison, WI) and CD58 shRNA or control constructs for 48 h prior to harvesting for analysis. The Dual luciferase reporter system (Promega, #E195A) was used to measure intracellular luciferase activity.

### Sphere formation assay

HCC cells (4 × 10^3^ per well) were inoculated into 6-well ultra-low adherence culture plates and cultured in DMEM/F12 (Gibco) supplemented with 15 ng/ml IGF (Peprotech, New Jersey, USA), 20 ng/ml β-FGF (Peprotech), 20 ng/ml EGF (Peprotech), 4 mU/ml insulin, 1 × B27 (Gibco) and 4 µg/ml heparin (Invitrogen). The spheroids' numbers were counted and photographed under the microscope.

### Proliferation assays and colony formation

The Cell Counting Kit 8 (CCK8; Dojindo, Kumamoto, Japan) was used to identify cell proliferation. Cell growth was determined by absorbance at 450 nm using a microplate reader (Tecan Infinite 200 PRO).

Cells (4 × 10^3^) were placed on 6-well plates, where they were grown for 10–14 days to determine colony formation. After 4% paraformaldehyde fixation and 1.0% crystal violet staining, the cells were observed under the microscope.

### Cell migration and invasion assay

The cells were plated in 200 μl of serum-free media in the upper compartment of a 24-well transwell chamber (Chemicon, Temecula, CA, USA). The bottom well was supplemented with normal cell culture media. After incubation, cells treated with 4% paraformaldehyde were visualized by staining with crystal violet.

### Enzyme-linked immunosorbent assay for sCD58 in culture medium

The Human sCD58 enzyme-linked immunosorbent assay (ELISA) kit (Cat No EH1082) was purchased from Finn Biotech (Wuhan, China). The level of sCD58 in the culture medium was determined following the instructions provided by the manufacturer.

### Statistical analysis

The results were expressed as mean ± SD. GraphPad Prism 8 (GraphPad Software, San Diego, CA) was used to create the graphs and analyze the data. Student’s t-test and one-way ANOVA were used to determine significant differences. The correlation between CD58 expression and clinicopathological features was evaluated by χ^2^ test and survival time was assessed using Kaplan–Meier analysis. Data were deemed statistically significant if *p* < 0.05.

## Results

### CD58 was highly correlated with poor prognosis by analyzing different databases

**Fig. 1:**
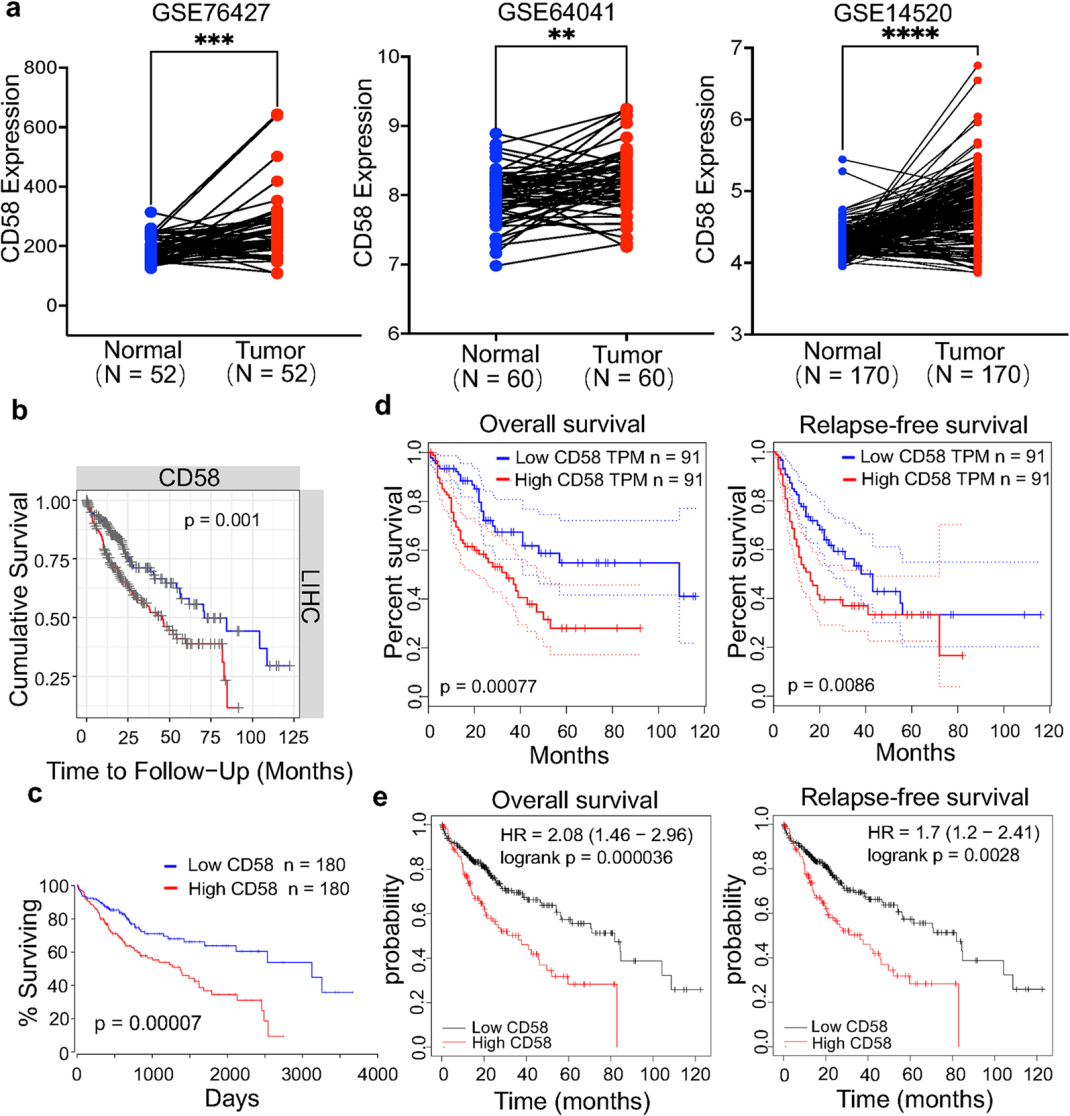
CD58 was highly correlated with poor prognosis by analyzing different databases. **a** CD58 mRNA expression in liver tumor tissues and normal liver tissues. **b**, **c** The TIMER (**b**) and OncoLnc databases (**c**) showed high CD58 expression was a poor prognostic indicator for HCC patients. **d**, **e** GEPIA (**d**) and Kaplan–Meier (**e**) database analysis revealed the relationship of CD58 with OS and disease-free survival in HCC

To examine the expression and function of CD58 in HCC progression, we analyzed CD58 expression in several GEO databases and observed that liver tumor tissues had greater levels of CD58 mRNA expression than normal liver tissues (Fig. 1a). Applying bioinformatics databases such TIMER, OncoLnc, GEPIA, and Kaplan–Meier plotter, we then assessed the predictive significance of CD58 expression in HCC patients. The overall survival (OS) of patients with elevated CD58 expression was significantly shortened, according to the TIMER and OncoLnc databases (*p* = 0.001 and *p* = 0.00007, respectively) (Fig. 1b and c). Analysis from GEPIA and Kaplan–Meier plotter databases, found CD58 expression was not only associated with OS (*p* = 0.00077 and *p* = 0.000036, respectively) (Fig. 1d and e, left panel), but also strongly correlated with Disease-free survival (*p* = 0.0086 and *p* = 0.0028) (Fig. 1d and e, right panel). Thus, CD58 may be a poor prognostic factor in HCC.

### Elevation of CD58 expression in HCC patients and predicted poor prognosis

**Fig. 2:**
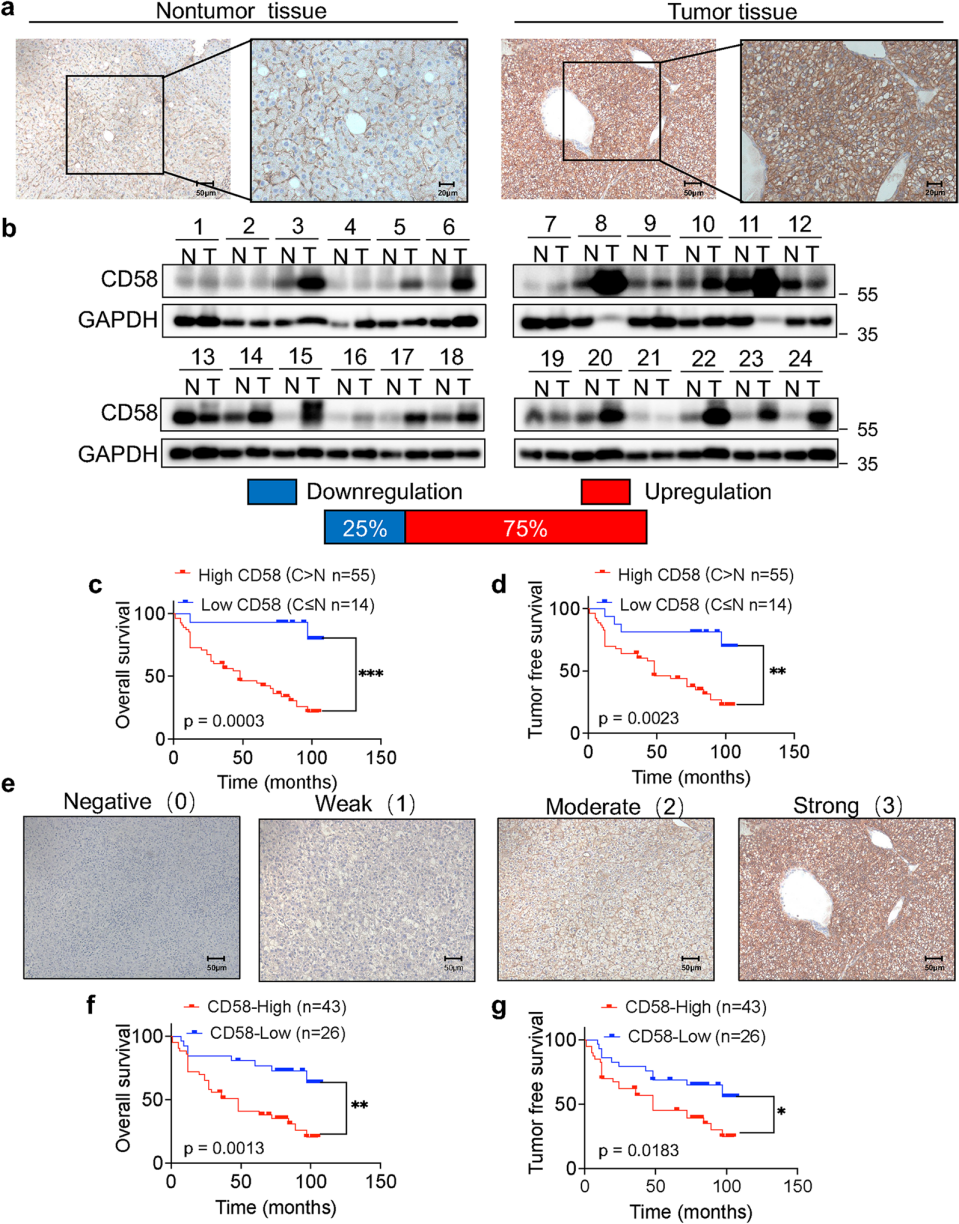
Elevation of CD58 expression in HCC patients and predicted poor prognosis. **a** Typical IHC pictures of CD58 in HCC patient tissues and surrounding non-tumor tissues. **b** The expression of the CD58 protein in pairs of HCC and comparable non-tumor tissues from 24 patients were examined by western blotting. **c, d** Kaplan Meier analysis of overall survival (**c**) and tumor-free survival (**d**) in 69 patients based on the level of CD58 expression. Define C > N as high CD58 expression and C ≤ N as low CD58 expression. **e** Different CD58 staining intensity scores in tumor tissue. Cell staining is classified as 0–3 (10 × original magnification). **f**, **g** Kaplan Meier analysis of overall survival (**f**) and tumor-free survival (**g**) in 69 patients based on the CD58 expression level in tumor tissues. An IHC score > 5 was considered as CD58-High expression, while others were CD58-Low expression

To further confirm the above observations, we gathered 69 pairs of clinical HCC patient tissues and surrounding non-tumor tissues to test the protein levels of CD58 by immunohistochemistry. Indeed, 79.7% (55/69) of HCC patient tissues showed higher CD58 protein expression levels than adjacent non-tumor tissues (Fig. 2a). Consistently, in another set of 24 paired HCC tissues, western blotting analysis revealed that CD58 protein levels were elevated in 75% (18/24) of HCC tissues (Fig. 2b). Based on immunohistochemistry-detected levels of CD58 protein expression, we analyzed the correlation between clinicopathological features and CD58 expression levels. The research showed that male patients (44/48) had higher levels of CD58 than female patients (11/21). High CD58 expression was substantially linked to large tumor size, poor differentiation, satellite focus, and vascular invasion (*p* < 0.05, Table 1). Subsequently, Kaplan–Meier analysis showed the OS (*p* = 0.0003, Fig. 2c) and tumor-free survival (*p* = 0.0023, Fig. 2d) of patients with high CD58 expression were significantly inferior to those with low CD58 expression. Furthermore, the Cox regression analysis identified satellite focus and CD58 were significant prognostic factors for HCC patients (*p* = 0.014 and 0.044, respectively) (Table 2).

**Table 1:** Association between CD58 expression and clinicopathological factors of 69 patients in HCC

Clinicopathological factors	High CD58 (n = 55)	Low CD58 (n = 14)	p value
Age			
≥ 60	40	8	0.2579
< 60	15	6	
Sex			
Male	44	4	0.0002***
Female	11	10	
Tumor size			
> 5 cm	27	2	0.0185*
≤ 5 cm	28	12	
Differentiation level			
Low/Middle	48	8	0.0101*
High	7	6	
AFP (μg /L)			
< 400	37	11	0.4120
≥ 400	18	3	
Satellite focus			
With	18	0	0.0128*
Without	37	14	
Tumor thrombus			
With	28	2	0.0136*
Without	27	12	
Liver cirrhosis			
With	21	4	0.5042
Without	34	10	
Chronic hepatitis			
With	18	4	0.9999
Without	37	10	

*AFP* Alpha-fetoprotein

*p < 0.05; **p < 0.01, ***p < 0.001

**Table 2:** Cox regression analysis of prognosis in 69 HCC patients

Factors	SE	p value	HR	95%CI	
				Lower	Upper
CD58 expression					
High (n = 55) versus low (n = 14)	0.773	0.044*	4.762	1.046	21.668
Age					
< 60 versus ≥ 60	0.015	0.224	1.018	0.989	1.048
Sex					
Male versus female	0.480	0.240	1.759	0.686	4.509
Tumor size					
≤ 5 cm versus > 5 cm	0.384	0.177	1.679	0.791	3.565
Differentiation level					
Low/Middle versus high	0.522	0.915	0.946	0.340	2.631
AFP (μg /L)					
< 60 versus ≥ 60	0.483	0.673	0.831	0.352	1.962
Satellite focus					
With versus without	0.366	0.014*	2.466	1.203	5.053
Tumor thrombus					
With versus without	0.391	0.146	1.767	0.821	3.807
Liver cirrhosis					
With versus without	0.369	0.995	0.882	0.428	1.816

*HR* hazard ratio, *CI* confidence interval

*Statistically significant, p < 0.05

To further determine the clinical significance of CD58, we used an objective IHC scoring method to assess and confirm the expression level of CD58 in tumor tissues (Fig. 2e). Samples with an IHC staining score of 0–5 were regarded as CD58-Low expression, whereas 6–7 were deemed as CD58-High expression. According to the survival analysis, HCC patients with CD58-High expression had substantially lower overall survival times (Fig. 2f, p = 0.0013) and tumor-free survival times (Fig. 2g, p = 0.0183) than those with CD58-Low expression. As shown in Table 3, the CD58-High expression group exhibited poor differentiation, larger tumor size, and vascular invasion. In addition, CD58 expression levels were higher in male or relatively elderly patients than those in female or relatively younger groups.

**Table 3:** Correlation between CD58 expression in tumor tissues and clinicopathological factors of 69 patients in HCC

Clinicopathological factors	CD58-High (n = 43)	CD58-Low (n = 26)	p value
Age			
≥ 60	38	10	< 0.0001***
< 60	5	16	
Sex			
Male	40	8	< 0.0001***
Female	3	18	
Tumor size			
> 5 cm	22	7	0.0481*
≤ 5 cm	21	19	
Differentiation level			
Low/Middle	38	18	0.0488*
High	5	8	
AFP (μg /L)			
< 400	29	19	0.6220
≥ 400	14	7	
Satellite focus			
With	13	5	0.3132
Without	30	21	
Tumor thrombus			
With	23	7	0.0313*
Without	20	19	
Liver cirrhosis			
With	15	16	0.7645
Without	28	10	
Chronic hepatitis			
With	14	18	> 0.8772
Without	29	8	

*AFP* Alpha-fetoprotein

*p < 0.05; **p < 0.01, ***p < 0.001

### CD58 promotes the proliferation of HCC cells in vitro and in vivo

**Fig. 3:**
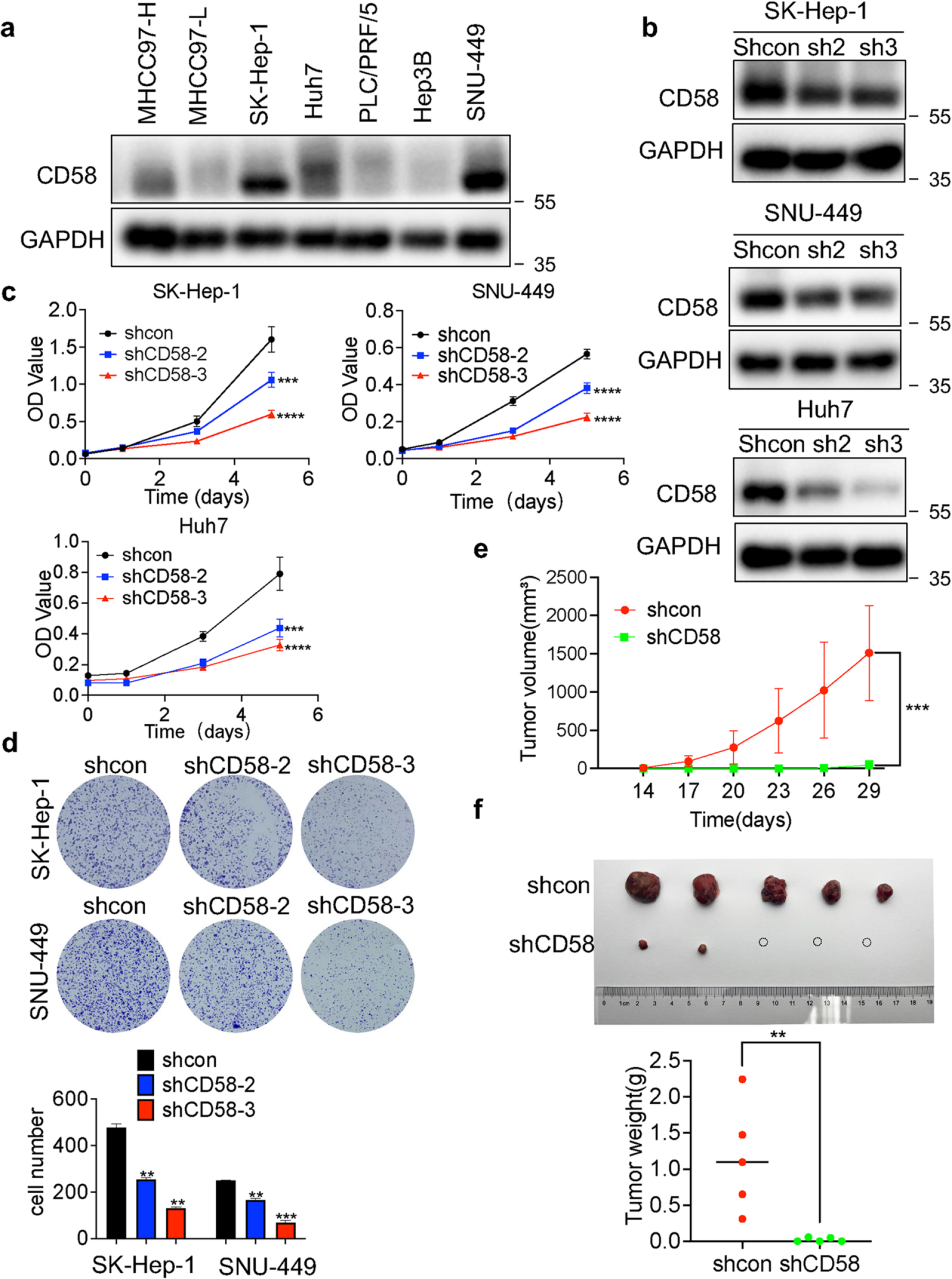
CD58 promotes the proliferation of HCC cells in vitro and in vivo. **a** CD58 protein levels in seven HCC cell lines were measured by western blotting. **b** Western blotting analysis of CD58 knockdown efficiency. **c**, **d** CCK-8 assay (**c**) and colony formation (**d**) analysis were conducted to determine the impact of CD58 depletion on the proliferation of SK-Hep-1, SNU-449, and Huh7 cells. **e** Huh7 cells expressing control (shcon) or CD58 shRNA (shCD58) were used for in vivo tumorigenesis. Tumor volume at indicated time (days) after tumor inoculation. **f** Tumors images and weight at day 29 after tumor inoculation

To investigate the biological functions of CD58, we first inspected the levels of CD58 protein (Fig. 3a) in seven HCC cell lines (MHCC97-H, MHCC97-L, SK-Hep-1, Huh7, PLC/PRF/5, Hep3B, SNU-449). Noteworthy, CD58 showed elevated expression in SK-Hep-1 and SNU-449 cells. Then, two shRNAs were used to knockdown two independent loci of CD58 in SK-Hep-1, SNU-449, and Huh7 cells, and the expression of CD58 in these cells was verified using a western blotting assay (Fig. 3b). CCK8 assay (Fig. 3c) and colony formation analysis (Fig. 3d) showed that CD58 knockdown inhibited the proliferation of HCC cells. We further investigated whether CD58 silencing has anti-tumor effects in vivo, Huh7 cells with or without CD58-knockdown were injected subcutaneously into the right and left flanks of nude mice. Tumors derived from CD58 stable-knockdown Huh7 cells grew more slowly and were significantly smaller than tumors from control Huh7 cells, as determined by the quantification of tumor volume (Fig. 3e) and tumor weight (Fig. 3f).

### CD58 and sCD58 increases HCC cell migration and invasion

**Fig. 4:**
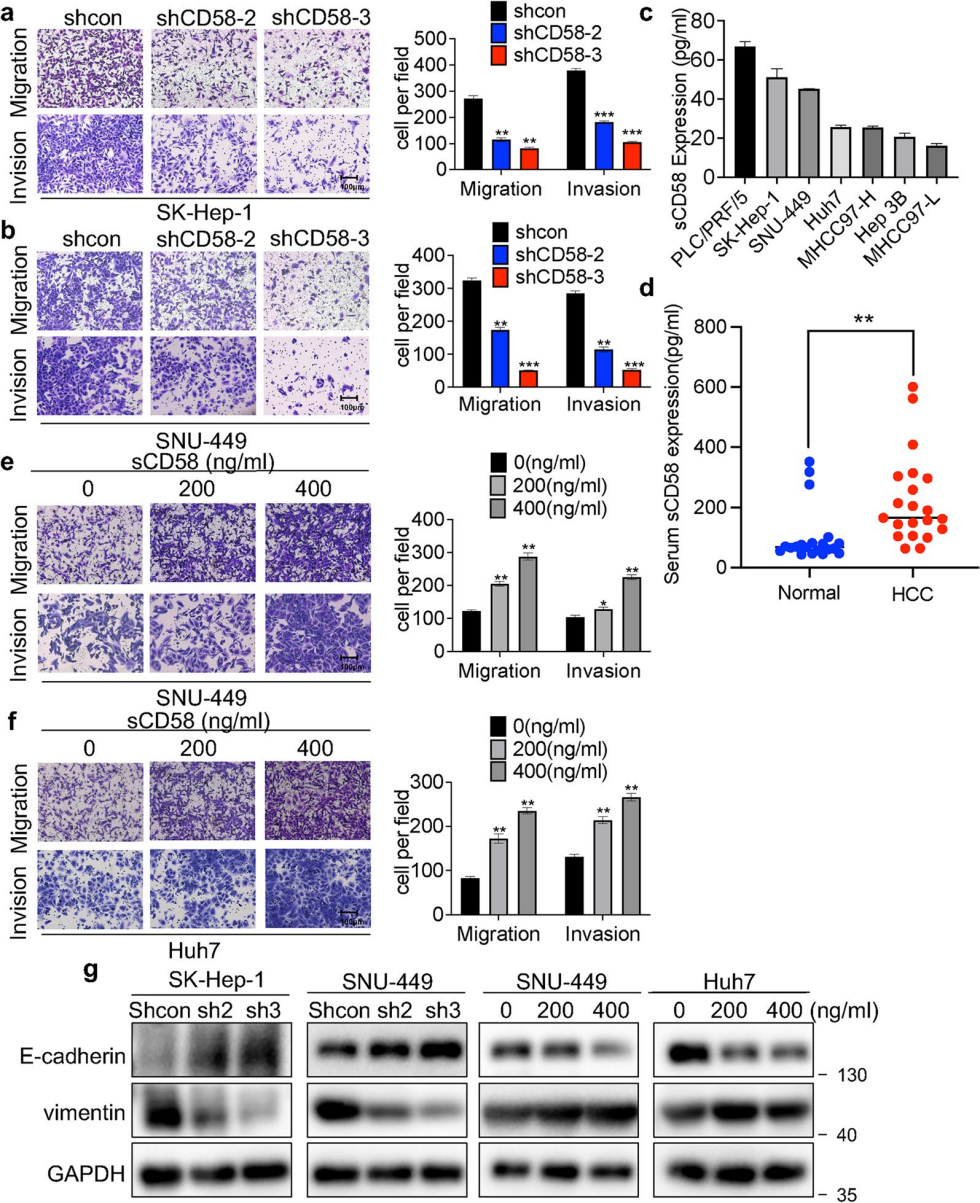
CD58 and sCD58 increase HCC cell migration and invasion. **a**, **b** The left panel displays typical pictures of migration (**a**) and invasion (**b**) assays for CD58 knockdown and control HCC cells, while the right panel displays the statistical results of Transwell assays. Five randomly chosen single fields of vision were used to count the cells under the microscope at 10 × magnification. **c**, **d** ELISA was used to measure the concentration of sCD58 in HCC cell supernatants (**c**) and serum of HCC patients and healthy individuals (**d**). **e**, **f** Transwell assays were used to assess the migration (**e**) and invasion (**f**) capabilities of HCC cells that had been incubated with various concentrations of sCD58. **g** Western blotting analysis of E-Cadherin and vimentin protein levels in the indicated cells (n = 3)

Based on the clinical significance of CD58 in promoting HCC metastasis, we further investigated the effect of CD58 on the metastatic ability of HCC cells using the Transwell assay. The migration and invasion ability in SK-Hep-1 cells were significantly decreased by the knockdown of CD58 (Fig. 4a). Similar reduced metastatic capacity was observed in SNU-449 cells as well (Fig. 4b).

Next, we examined the level of sCD58 in the supernatant of HCC cell lines and serum of HCC patients and healthy individuals by ELISA. As expected, all HCC cell lines secreted sCD58, with PLC/PRF/5 and SK-Hep-1 having sCD58 levels greater than 50 pg/ml (Fig. 4c), and serum levels of sCD58 were significantly elevated in HCC patients (Fig. 4d). Interestingly, sCD58 increased SNU-449 and Huh7 cell migration and invasion in a dose-dependent manner (Fig. 4e and f). As shown in Fig. 4g, the knockdown of CD58 in SNU-449 and SK-Hep-1 cells raised the expression of E-cadherin while downregulating the expression of vimentin. In contrast, SNU-449 and Huh7 cells showed the opposite result in response to sCD58. Overall, these findings demonstrate that CD58 and sCD58 modulate HCC cell migration and invasion in HCC cells.

### CD58 and sCD58 induced cancer cell stemness of HCC cells

**Fig. 5:**
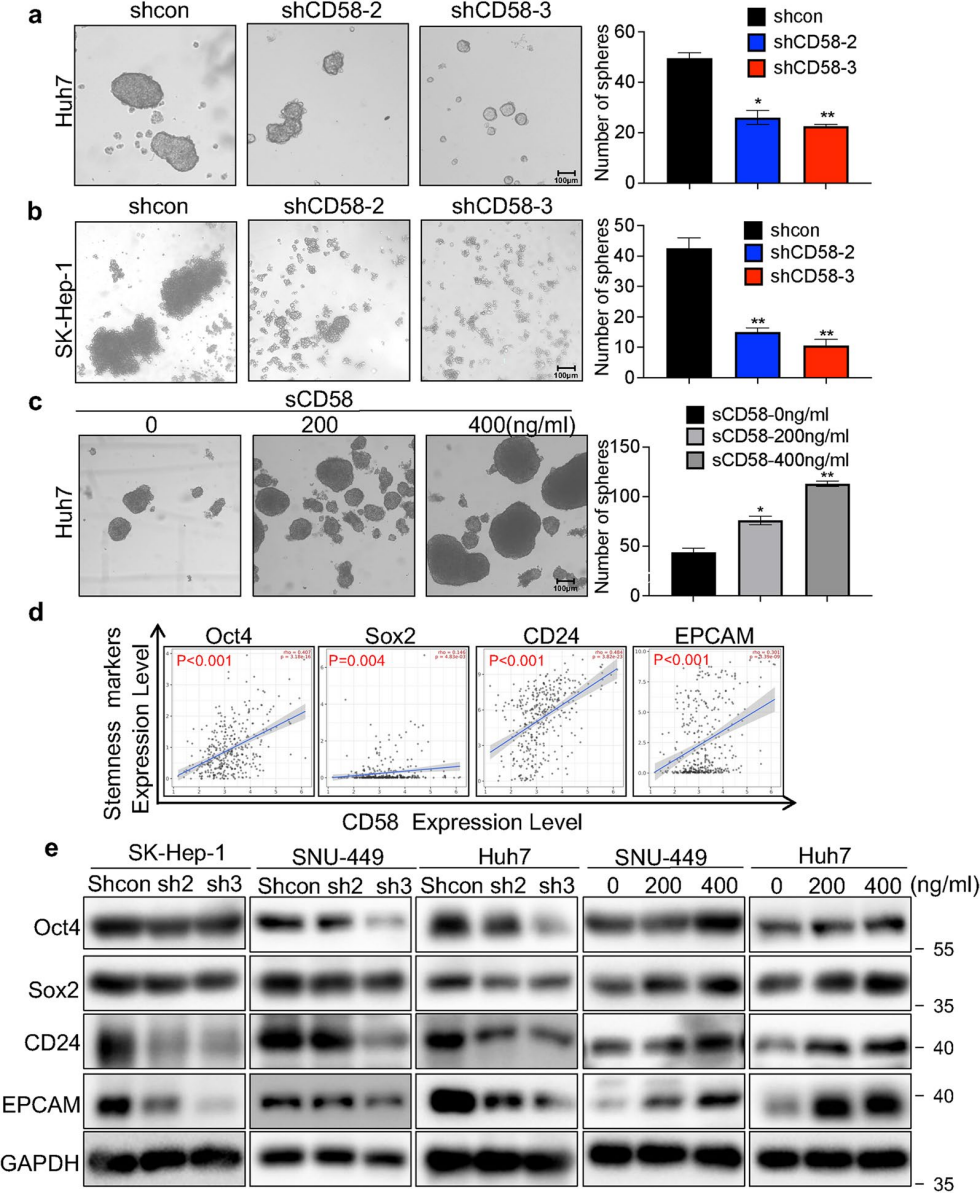
CD58 and sCD58 induced cancer cell stemness of HCC cells. **a**, **b** CD58 knockdown impaired sphere-forming capacity in Huh7 (**a**) and SK-Hep-1 (**b**) cells. **c** Effects of sCD58 on sphere-forming capacity in Huh7 cell. **d** Correlation of CD58 with cancer stemness markers by analyzing the TIMER database. **e** Western blotting analysis of Oct4, Sox2, CD24, and EPCAM protein levels in the indicated cells (n = 3)

Due to the poor sphere-forming ability of SNU-449 cells, Huh7 cells were used in the spheroid formation assay. We found that CD58 depletion greatly reduced the number of spheres in Huh7 cells (Fig. 5a). Similarly, a substantial decrease in the ability to form spheres was observed in CD58-knockdown SK-Hep-1 cells (Fig. 5b). On the contrary, sCD58 promoted spheroid formation in Huh7 cells in a concentration-dependent manner (Fig. 5c). The counting of the number of spheres provided evidence to support the findings. To investigate whether CD58 induces pluripotency of HCC cells, we then analyzed the correlation between CD58 and the stemness markers, including Oct4, Sox2, CD24, and EPCAM in the TIMER database. Our analysis revealed that CD58 is positively correlated with the above markers (Fig. 5d). Moreover, these protein levels in HCC cells were reduced markedly after CD58 knockdown, as evidenced by Western blotting (Fig. 5e). In contrast, sCD58 achieved the opposite effect (Fig. 5e). These data demonstrate that CD58 and sCD58 regulate the self-renewal and pluripotency in HCC cells.

### CD58 activates the Wnt/β-catenin signaling pathway in HCC cells

**Fig. 6:**
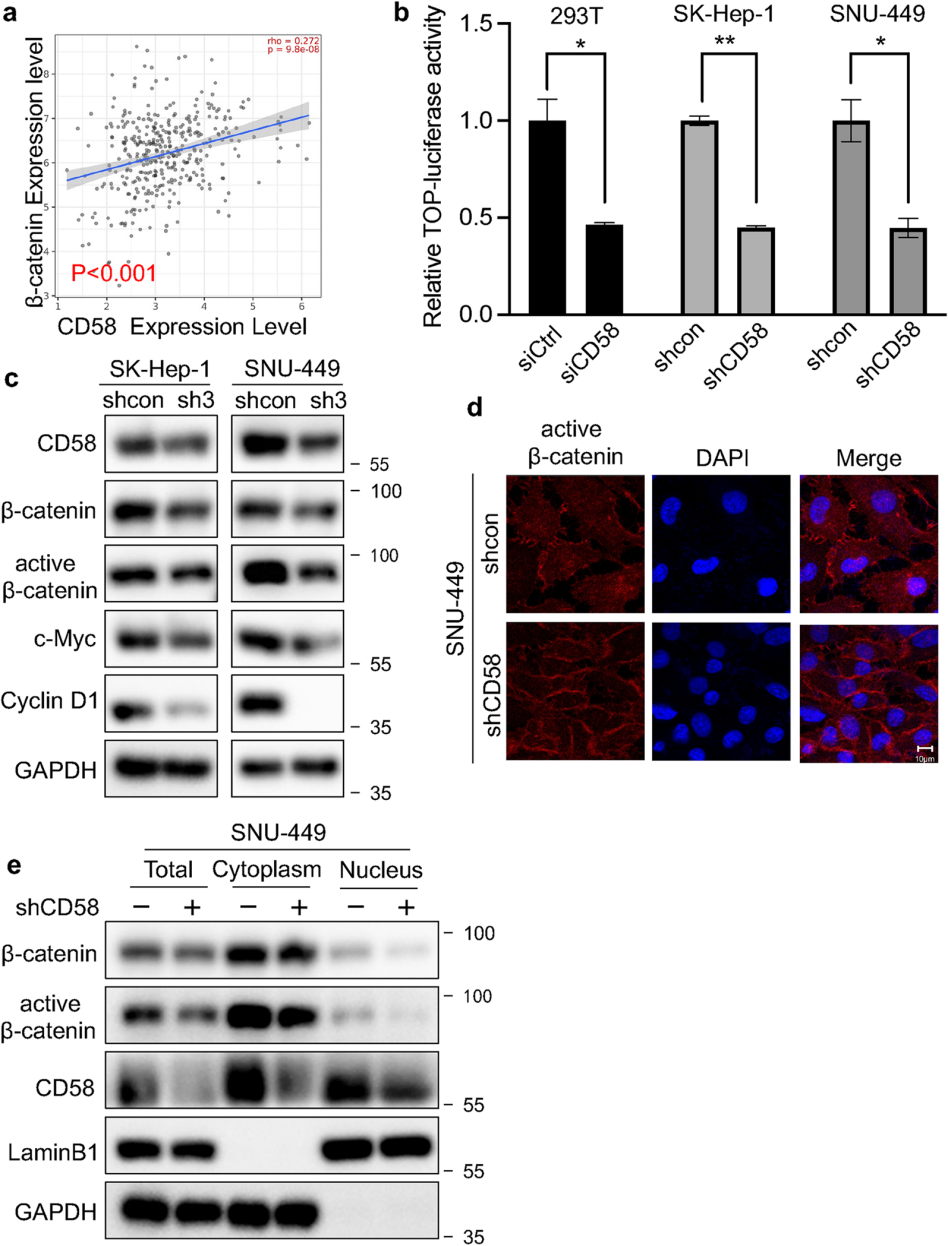
CD58 activates the Wnt/β-catenin signaling pathway in HCC cells. **a** The association between CD58 and β-catenin from the TCGA database. **b** TOP luciferase activity assay revealed the effect of CD58 silencing on TCF/LEF-mediated transcription activity. **c** Western blotting analysis of the levels of CD58, total β-catenin, active β-catenin (S33/37/T41), Cyclin D1, and c-Myc protein (n = 3). **d** Immunofluorescence analysis of the localization of active β-catenin. **e** A subcellular fractionation assay was performed to isolate the nuclear and cytoplasmic fractions and evaluated the expression of active and total β-catenin (n = 2)

β-catenin is thought to be a central molecule in the Wnt/β-catenin signaling pathway and is essential for cancer cell metastasis and self-renewal [25]. We first identified a positive association between CD58 and β-catenin in HCC samples from the TCGA database (Fig. 6a). Then, we investigated the transcriptional activity of β-catenin using a Top flash reporter assay. The results indicated the transactivation of TCF reporter was impaired in CD58 knockdown HCC cells and 293 T cells relative to their corresponding controls, respectively, indicating CD58 depletion reduced β-catenin/TCF-LEF-mediated transcriptional activity (Fig. 6b). Next, we analyzed the protein expression of β-catenin and some transcriptional targets of the Wnt/β-catenin signaling pathway, such as Cyclin D1 and c-Myc. As shown in Fig. 6c, the protein expression levels of total β-catenin and active β-catenin were significantly decreased in CD58 knockdown HCC cells, accompanied by reduced expression of Cyclin D1 and c-Myc. An Immunofluorescence experiment was performed to detect the nuclear localization of active β-catenin. As can be appreciated visually, CD58 knockdown markedly reduced nuclear accumulation of active β-catenin (Fig. 6d). We also examined the active and total β-catenin protein levels in nuclear and cytoplasmic fractions in HCC cells. Consistently, the active and total β-catenin protein expression levels were reduced in nuclear and cytoplasmic fractions of CD58 knockdown HCC cells relative to control cells (Fig. 6e). Collectively, these findings implicate that CD58 promotes the transcriptional activation of β-catenin.

### CD58 regulates β-catenin activity by modulating AKT/GSK-3β signaling in HCC cells

**Fig. 7:**
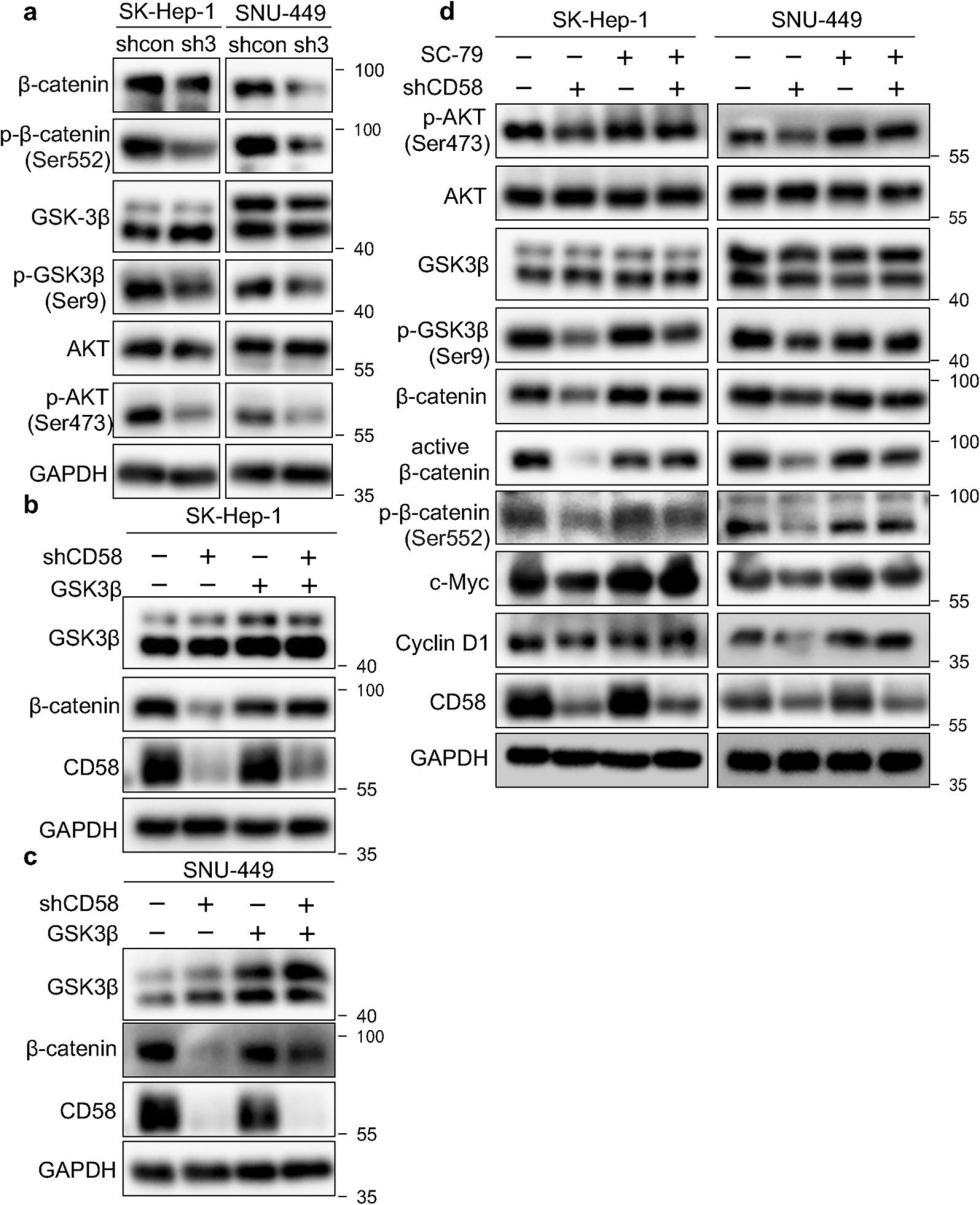
CD58 regulates β-catenin activity by modulating AKT/GSK-3β signaling in HCC cells. **a** Western blotting analysis of the protein levels of β-Catenin, p-β-catenin (Ser552), GSK-3β, p-GSK-3β(Ser9), AKT, and p-AKT(Ser473) (n = 3). **b**, **c** Protein expression of the stable CD58-knockdown SK-Hep-1 (**b**) and SNU-449 (**c**) cells transfected with GSK-3β were detected by western blotting with corresponding antibodies (n = 2). **d** SK-Hep-1 and SNU-449 cells with stable CD58 knockdown were incubated with SC-79 for 24 h, followed by western blotting with the corresponding antibodies (n = 3)

The process of β-catenin degradation is facilitated by the phosphokinase activity of GSK-3β. This activity is hindered by the phosphorylation of activated phospho-AKT, which is a typical oncogenic kinase that significantly contributes to tumor progression [26]. Therefore, using western blotting, we further evaluated the protein levels of the AKT, GSK-3β, β-Catenin, and p-β-Catenin (Ser552) that is phosphorylated by AKT signaling. AKT and GSK-3β protein levels were unaffected by CD58 knockdown, while levels of p-AKT, p-GSK-3β, and β-catenin were reduced (Fig. 7a). GSK-3β was transfected into CD58-knockdown cells and control cells to further investigate if CD58 performs its role via GSK-3β. The results indicated that GSK-3β overexpression abolished the inhibitory effect of CD58 knockdown on β-catenin protein (Fig. 7b and c). Thus, CD58 inhibited the phosphorylation and degradation of β-catenin mediated by GSK-3β. Moreover, treatment of CD58 knockdown HCC cells with Akt activator (SC-79) partially reverses their inhibition of phosphorylated GSK-3β and AKT. Akt activator also impaired the inhibition of the protein expression of total β-catenin, p-β-catenin, active β-catenin, c-Myc, and Cyclin D1 resulting from CD58 depletion (Fig. 7d). These data suggest CD58 can mediate the inactivation of GSK-3β by increasing AKT phosphorylation, thereby reducing β-catenin phosphorylation and activating β-catenin transcriptional activity.

### CD58 exerts the tumor-promotion role in HCC cells via AKT/GSK-3β/β-catenin signaling

**Fig. 8:**
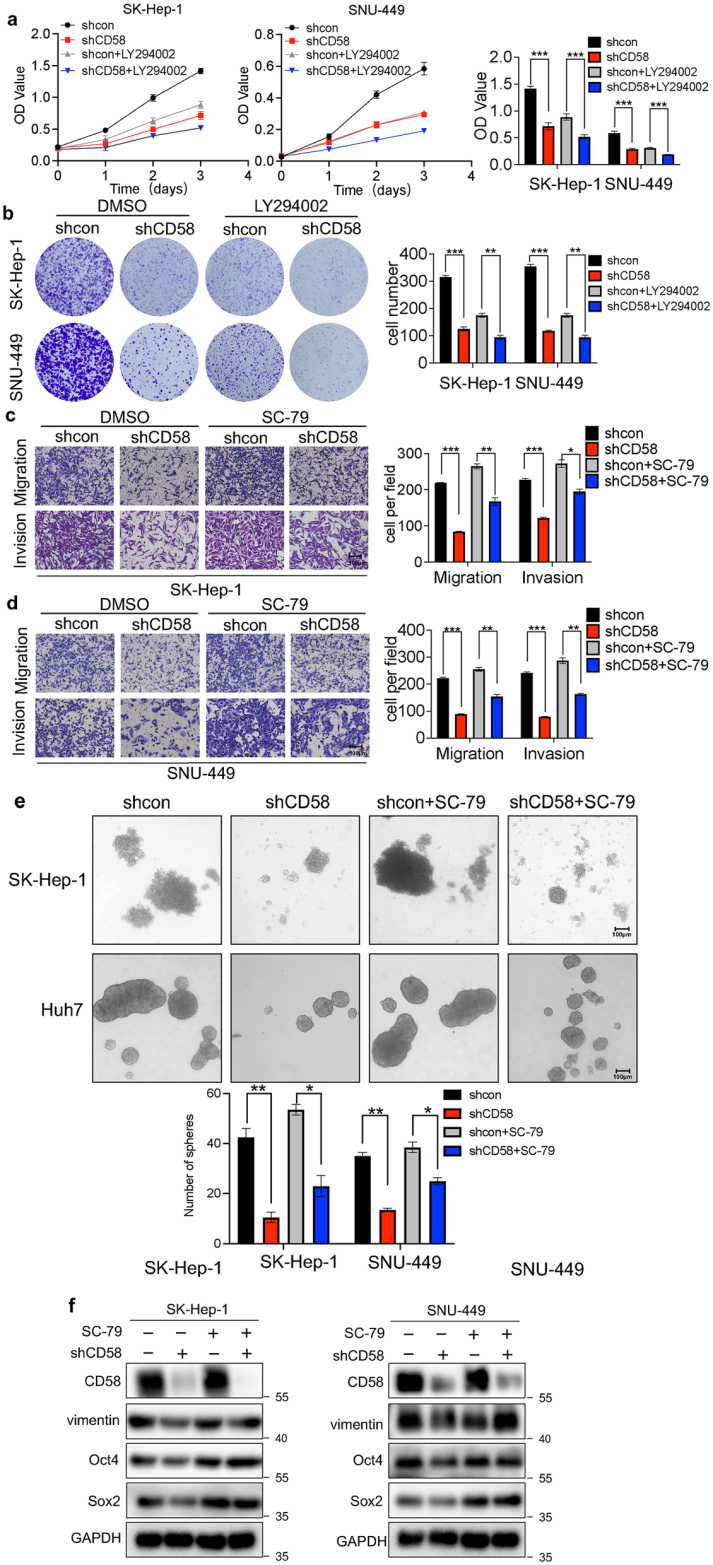
AKT/GSK-3β/β-Catenin pathway regulates the tumor-promotion effect of CD58 in HCC cells. **a**, **b** CCK-8 assay (**a**) and colony formation (**b**) analysis indicated that LY294002 treatment eliminated CD58 knockdown-mediated suppression of HCC cell growth. **c**, **d** SC-79 attenuated HCC cell’s inhibition of migration and invasion induced by CD58 knockdown in SK-Hep-1 (**c**) and SNU-449 (**d**) cells. **e** Spherical formation experiments showed that SC-79 treatment eliminated the inhibition of CD58 silencing on the stemness of SK-Hep-1 and Huh7 cells. **f** SK-Hep-1 and SNU-449 cells with stable CD58 depletion were incubated with SC-79 for 24 h, followed by western blotting with the corresponding antibodies (n = 3)

To determine whether CD58 silencing exerts an antitumor effect via the inhibition of the AKT/GSK-3/-Catenin pathway, CD58 knockdown HCC cells were treated with Akt activator (SC-79) and AKT inhibitor (LY294002). As expected, the treatment of LY294002 abrogated CD58 silencing-mediated inhibition of HCC cell proliferation (Fig. 8a and b). Meanwhile, Akt activator treatment dramatically recovered CD58 knockdown-induced diminishment of cell migration and invasion. Consistently, the quantification of migratory and invasive cells confirmed these observations (Fig. 8c and d). In addition, treatment of CD58 knockdown Huh7 and SK-Hep-1 cells with SC-79 partially reversed their spheroid-forming ability impaired by CD58 silencing (Fig. 8e). Western blotting assays further confirmed these findings, exposure to Akt activator resulted in the upregulation of the protein levels of vimentin, Sox2 and Oct4 (Fig. 8f). These results indicated AKT/GSK-3β/β-Catenin signal pathway is engaged in CD58-induced proliferation, metastasis and stemness of HCC cells.

### sCD58 activates the AKT/GSK-3β/β-catenin pathway in HCC cells

**Fig. 9:**
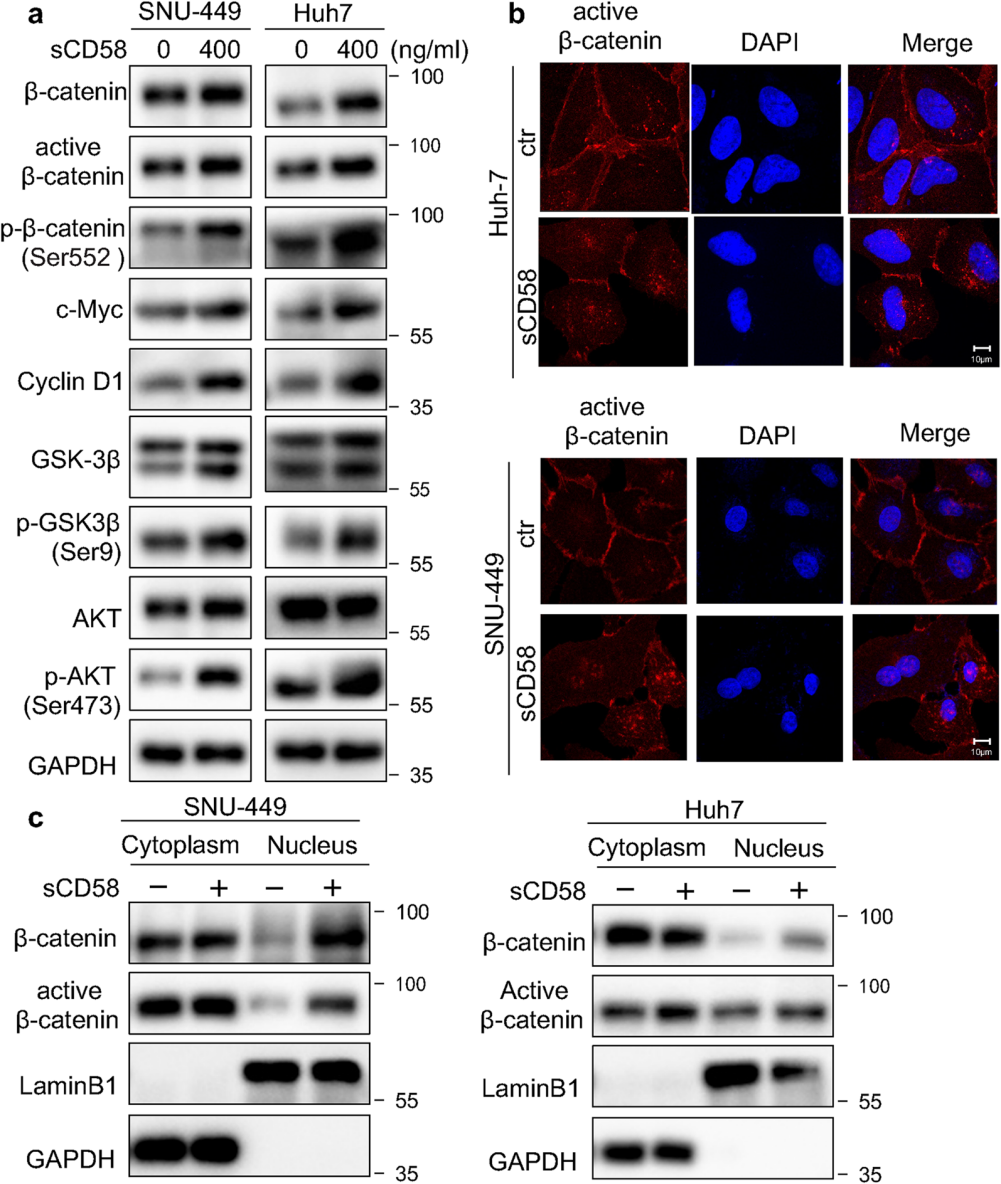
sCD58 activates the AKT/GSK-3β/β-Catenin pathway in HCC cells. **a** Representative western blotting results of AKT/GSK-3β/β-catenin pathway-related proteins (n = 3). **b** Immunofluorescence analysis of the localization of active β-catenin. **c** Subcellular fractionation analysis of the expression of total and active β-catenin in the nuclear and cytoplasmic fractions (n = 2)

We examined whether sCD58 modulated AKT/GSK-3β/β-catenin signaling to influence the biological phenotype in HCC cells. Our research demonstrated that treatment with sCD58 considerably raised the protein levels of p-AKT, p-GSK-3β, and active β-catenin (Fig. 9a). The protein expression of Cyclin D1 and c-Myc were also increased (Fig. 9a). Furthermore, Immunofluorescence experiments and subcellular fractionation showed that sCD58 increased nuclear accumulation of active β-catenin, respectively (Fig. 9b and c).

## Discussion

To our knowledge, this is the first research to explore the potential function and mechanisms of sCD58 and CD58 in HCC. We found sCD58 was released by HCC cells, and higher levels of sCD58 in HCC patient serum than in healthy controls. Also, CD58 was elevated in HCC patient tissues and its expression relates to patient prognosis. Further study demonstrated that CD58 and sCD58 exert their pro-tumor effects by activating the AKT/GSK-3β/β-Catenin pathway.

There is accumulating evidence that cancer stem cells (CSCs) are present in most solid malignancies, including HCC [27]. CSCs are a small fraction of tumor cells that are capable of self-renewal and differentiation, inducing tumor progression, metastasis, and chemotherapy resistance [28]. Liver CSCs are enriched in certain defined markers, including EPCAM, CD24, CD133, Oct4 and Sox2, etc. [29, 30]. Our study showed that CD58 increased the expression levels of pluripotency markers, such as Oct4, Sox2, CD24 and EPCAM, as well as the sphere-forming ability of HCC cells. In colon cancer cells, CD58 was reported to be a strong surface marker for colorectal tumor-initiating cells and promoted the cells’ self-renewal ability by upregulating the Wnt/β-catenin pathway [16]. Considering that the Wnt/β-catenin pathway plays an integral role in generation and maintaining stemness of CSC [31], we speculate that they may share a common self-renewal regulatory mechanism in CRC and HCC. As expected, this study revealed that knockdown of CD58 in HCC cells significantly reduced the expression of Wnt/β-catenin target proteins and inhibited TCF/LEF-mediated transcriptional activity, indicating that CD58 activates the Wnt/β-catenin pathway.

The glycosylphosphatidylinositol (GPI)-anchored and transmembrane forms are two isoforms of CD58 [32], the former localized in lipid rafts to strengthen adhesions and mediate signal transduction while the latter in non-raft membrane domains involved in signaling [33, 34]. Through cross-linking with CD58, the transmembrane isoform triggers tyrosine phosphorylation of multiple proteins independent of the GPI-anchored isoform, including Akt/PKB (Ser473 and Thr308) [34]. Moreover, Neural cell adhesion molecule (CD56), also an immune adhesion molecule, was shown to activate the Wnt/β-catenin and PI3K-AKT signaling pathways to induce osteoblast differentiation [35]. In this study, we also demonstrated CD58 activates the Wnt/β-catenin and PI3K-AKT signaling pathways. As we all know, GSK3β is the linkage between PI3K/AKT pathway and Wnt/β-catenin pathway and is essential for the stability of β-catenin in the cytoplasm [36, 37]. We found that activation of Akt or GSK-3β reverses CD58 knockdown-mediated suppression of the Wnt/β-catenin pathway. In addition, SC-79 or LY294002 abolished the inhibitory effect of CD58 silencing on the proliferation, metastasis, and self-renewal ability of HCC cells. These results suggest that CD58 induces the malignant phenotype of HCC through the AKT/GSK3β/β-catenin pathway.

Regarding the mechanism of sCD58 release, it has not been well understood, and cleavage of membranous CD58 is thought to be the source of sCD58 [11]. sCD58, as a biological immunomodulator, interferes with cell adhesion and recognition in vivo [7, 38], but also enhances T-cell growth and activation [38]. Large amounts of sCD58 released by melanoma inhibit cellular immune responses and tumor cell lysis, reducing the sensitivity of immunotherapy [39]. Notably, our study found that sCD58 is involved in regulating the metastasis and stemness of HCC. Similar studies were conducted in sCD146 [40] and secretory clusterin (sCLU) [41]. sCD146 secreted by CD146-positive tumors mediates important pro-angiogenic and pro-tumoral effects [40], while sCLU promotes chemoresistance, metastasis and CSC phenotype in HCC by activating the AKT/GSK3β/β-catenin axis [41]. Although we have demonstrated that CD58 and sCD58 activate AKT signaling pathway, it is not clear how sCD58 increases the phosphorylation level of AKT. Interestingly, LRIG3 and sLRIG3 can regulate the MET/PI3K/Akt pathway in GBM [42], and further study found sLRIG3 also binds to the transmembrane protein NETO2 to activate the NF-kB pathway in GBM [43]. TELO2, a cofactor of phosphatidylinositol 3-kinase-related kinases, has been reported to bind to RICTOR as the mTORC2 complex and promote CRC cell progression through the AKT pathway [44]. Ivermectin, an inhibitor of the Wnt/β-catenin pathway, binds to TELO2 and mediates the aberrant expression of the AKT/mTOR and Wnt/β-catenin pathways [45]. Moreover, recombinant human CHI3L1 (rhCHI3L1) interacts with CD44, activates Erk and Akt signaling, and notably triggers the β-catenin pathway [46]. Therefore, we speculate that the activation of AKT pathway by sCD58 may be attributed to its binding to cell membrane proteins.

The previous study suggested that sCD58 levels in the serum of HBV-infected patients are substantially higher than in the normal population and correlate with disease severity [47]. The majority of HCC occurs in the presence of chronic liver disease and cirrhosis, for which hepatitis is the leading cause [48]. In this study, we found that sCD58 existed in HCC cells supernatant, and patients with HCC have higher serum levels of sCD58 compared to healthy individuals. However, due to the limited sample size of HCC serum, the effect of sCD58 on the prognosis of HCC patients is unclear, and whether sCD58 can increase the risk of HCC development remains to be further elucidated.

In conclusion, we demonstrated elevated expression of CD58 in HCC and elucidated its clinical significance. Both CD58 and sCD58 exert oncogenic effects in HCC through activation of the AKT/GSK-3β/β-Catenin pathway. Considering the potential role and mechanisms associated with CD58 and sCD58 in HCC, they may represent key prognostic markers and offer possibilities for HCC treatment.

## Data Availability

All data generated or analyzed during this study are included in this published article.

## Abbreviations

HCC: Hepatocellular carcinoma
GSK-3β: Glycogen synthase kinase (GSK)-3β
sCD58: A soluble form of CD58
DMEM: Dulbecco’s modified Eagle’s medium
Active β-catenin: Non-phosphorylated (active) β-catenin (S33/37/T41)
CSC: Cancer stem cells
TCGA: The cancer genome atlas
GEO: Gene expression omnibus
